# Vision without the Image

**DOI:** 10.3390/s16040484

**Published:** 2016-04-06

**Authors:** Bo Chen, Pietro Perona

**Affiliations:** Computation and Neural Systems, California Institute of Technology, 1200 E California Blvd, Pasadena, CA 91125, USA; bchen3@caltech.edu

**Keywords:** photon-counting sensors, visual recognition, low-light computer vision

## Abstract

Novel image sensors transduce the stream of photons directly into asynchronous electrical pulses, rather than forming an image. Classical approaches to vision start from a good quality image and therefore it is tempting to consider image reconstruction as a first step to image analysis. We propose that, instead, one should focus on the task at hand (e.g., detection, tracking or control) and design algorithms that compute the relevant variables (class, position, velocity) directly from the stream of photons. We discuss three examples of such computer vision algorithms and test them on simulated data from photon-counting sensors. Such algorithms work just-in-time, *i.e.*, they complete classification, search and tracking with high accuracy as soon as the information is sufficient, which is typically before there are enough photons to form a high-quality image. We argue that this is particularly useful when the photons are few or expensive, e.g., in astronomy, biological imaging, surveillance and night vision.

## 1. Introduction

Current computer vision algorithms start with a high-quality image as input. While such images may be acquired almost instantly in a well-lit scene, dark environments demand a significantly longer acquisition time. This long acquisition time is undesirable in many applications that operate in low-light environments: in biological imaging, prolonged exposure could cause health risks [1] or sample bleaching [2]; in autonomous driving, the delay that is imposed by image capture could affect a vehicle’s ability to stay on-course and avoid obstacles; in surveillance, long periods of imaging could delay response, as well as produce smeared images. When light is low, the number of photons per pixel is small and images become noisy. Computer vision algorithms are typically not designed to be robust *vis-a-vis* image noise, thus practitioners face an uneasy tradeoff between poor performance and long response times.

Novel sensor technology offers a new perspective on image formation: as soon as a photon is sensed it should be transmitted to the host Central Processing Unit (CPU), rather than wait until a sufficient number of photons has been collected to form good quality image. Thus an image, in the conventional sense, is never formed. Designs and prototypes of photon-counting image sensors, such as the quantum sensors [3], single-photon avalanche detectors [4], quanta image sensors [5,6], and the giga-vision camera [7], have been proposed recently. These sensors are capable of reliably detecting single photons, or a small number of photons. Instead of returning a high-quality image after a long exposure, photon-counting sensors report a stream of photon counts densely sampled in time.

Currently, the dominant use for photon-counting image sensors is image reconstruction [8]: the stream of photon counts is used to synthesize a high-quality image to be used in consumer applications or computer vision. However, the goal of vision is to compute information about the world (class, position, velocity) from the light that reaches the sensor. Thus, reconstructing the image is not a necessary first step. Rather, one should consider computing information directly from the stream of photons [9,10]. This line of thinking requires revisiting the classical image-based paradigm of computer vision, and impacts both the design of novel image sensors and the design of vision algorithms.

Computing directly from the stream of photons presents the advantage that some information may be computed immediately, without waiting for high-quality image to be formed. In other words, information is computed incrementally, enabling the downstream algorithms to trade off reaction times with accuracy. This is particularly appealing in low-light situations were photon arrival times are widely spaced. As the hardware for computation becomes faster, this style of computation will become practical in brighter scenes, especially when response times are crucial (e.g., in vehicle control).

Here we explore three vision applications: classification, search and tracking. In each application, we will propose an algorithm that makes direct use of the stream of photons, rather than an image. We find that each one of these algorithms achieves high accuracy with only a tiny fraction of the photons required for capturing high-quality images. We conclude with a discussion of what was learned.

## 2. Results

### 2.1. Simplified Imaging Model

Assume that the scene is stationary and photon arrival times follow a homogeneous Poisson process. Within an interval of length $\delta_{t}$, the observed photon count $X_{i}$ at pixel location *i* is subject to Poisson noise whose mean rate depends on maximum rate $\lambda_{max}\in R^{+}$, the true intensity at that pixel $I_{i}\in \left[0,1\right]$ and a dark current rate $\epsilon_{dc}\in \left[0,1\right]$ per pixel [8]:

(1) $$\begin{array}{cc} P(X_{i}=k) & =Poisson(k;\lambda_{max}\frac{\left(I_{i}+\epsilon_{dc}\right)}{\left(1+\epsilon_{dc}\right)}\delta_{t}) \end{array}$$

(a model including sensor read noise is described in Section 3.1).

**Figure 1:**
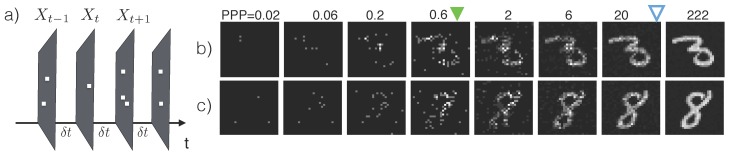
Synthetic low-light images. (**a**) A photon-counting sensor outputs a matrix of photon counts $X_{t}$ with a period of δt. (**b,c**) Sample synthetic low-light images from the Mixed National Institute of Standards and Technology (MNIST) dataset [11] used in the classification experiments (Section 2.2.2) with increasing average photons per pixel (PPP). PPP is proportional to the exposure time *t*. Blue hollow arrows indicate the median PPP required for the proposed algorithm (Section 2.2.1) to achieve the same error rate (0.7%) as a model trained and tested using images under normal lighting conditions with about $2^{7}\approx 10^{4}$ PPP. Green solid arrows indicate the median PPP required to to maintain error rates below 1%.

The sensor produces a stream of images $X_{1},X_{2},\ldots$, where $X_{t}\in N^{d}$ contains the photon counts from *d* pixel locations from the time interval $\left[\left(t-1\right)\delta_{t},t\delta_{t}\right]$ (Figure 1a). We use $X_{1:t}$ to represent the stream of inputs $\left\{X_{1},X_{2},\ldots ,X_{t}\right\}$.

When the illuminance of the environment is constant, the expected number of photons collected by the sensor grows linearly with the exposure time. Hence we use the number of photons per bright pixel (PPP) as a proxy for the exposure time *t*. PPP =1 means that the a pixel with maximum intensity has collected 1 photon. Additionally, since PPP is linked to the total amount of information content in the image regardless of the illuminance level, we will use PPP when describing the performance of vision algorithms. Figure 1b, c shows two series of inputs $X_{1:t}$ with increasing PPP.

### 2.2. Classification

Distinguishing objects of different categories hinges upon the extraction of “features”, which are structural regularities in pixel values such as edges, corners, contours, *etc*. For example, the key feature that set apart a handwritten digit “3” from a digit “8” (Figure 1b,c, last column) is the fact that a “3” has open loops and “8” has closed loops—This corresponds to different strokes on the left side of the digit. In normal lighting conditions, these features are fully visible, may be computed by, e.g., convolution with an appropriate kernel, and fed into a classifier to predict the category of the image.

In low light, classification is hard because the features are corrupted by noise. A closed contour may appear broken due to stochastically missing photons. The noise in the features in turn translates to uncertainties on the classification decision. This uncertainty diminishes as the exposure time increases. It is intuitive that a vision algorithm that is designed to compute from a minimal number of photons should keep track of said uncertainties, and dynamically determine the exposure time based on the desired accuracy.

In particular, one wishes to predict the category Y∈{1,2,…,C} of an image based on photon counts $X_{1:t}$. The predictions must minimize exposure time while being reasonably accurate, *i.e.*,

(2) $$\begin{array}{c} \min E\left[T\right]\;\;\;\;s.t.\;\;\;E\left[\hat{Y}\neq Y\right]\leq \gamma \end{array}$$

where *T* is a random variable denoting the exposure time required to classify an image, $\hat{Y}$ ∈ {1,2,…,C} is the prediction of the class label, *γ* is the maximum tolerable misclassification error, and the expectation is taken over all images in a dataset.

#### 2.2.1. Classification Algorithm

**Figure 2:**
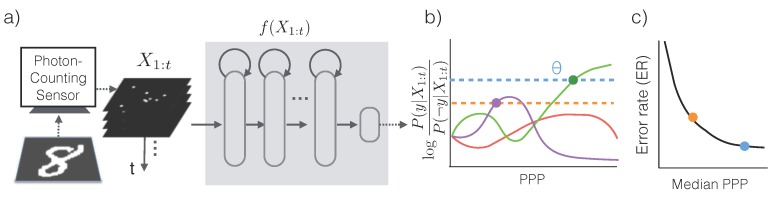
Low-light classification. (**a**) A sensor produces a stream of photon counts $X_{1:t}$, which is fed into a recurrent neural network to compute class conditional likelihood $f\left(X_{1:t}\right)\approx P\left(Y|X_{1:t}\right)$. (**b**) SPRT with three classes. Based on $f(X_{1:t})$, SPRT compares the class with the highest log likelihood ratio to a threshold *θ*. Two threshold options (dashed lines) corresponds to different decisions at different times (solid dots). (**c**) The threshold allows the system to traverse the error rate (ER) *versus* PPP curve to find the optimal operating point to minimize the cost function (Equation (2)). We use PPP instead of the exposure time *T* to measure speed as the former is more closely related to the information content in the photon stream. We use the median PPP instead of the mean because PPP follows a heavy-tailed distribution (Figure 3c) and median is more stable than the mean.

In order to make the most efficient use of photons, we first assume that a conditional probabilistic model $P(Y|X_{1:t})$ is available for any t≥0 (we will relax this assumption later) and for all possible categories of the input image. An asymptotically optimal algorithm that solves the problem described in Equation (2) is Sequential Probability Ratio Testing (SPRT) [12] (Figure 2a,b):

(3) $$\begin{array}{cc} & \text{Choose an appropriate error threshold}\;\;\;\theta \\ & c^{*}=\underset{c\in \{1,2,\ldots ,C\}}{\arg\max}\;P\left(Y=c|X_{1:t}\right)\\ & \left\{\begin{array}{cc} \text{report}\;\;\;Y=c^{*} & \text{if}\;\log\frac{P(Y=c^{*}|X_{1:t})}{P(Y\neq c^{*}|X_{1:t})}>\theta \\ \text{increase}\;\;\;t & \text{otherwise} \end{array}\right. \end{array}$$

**Figure 3:**
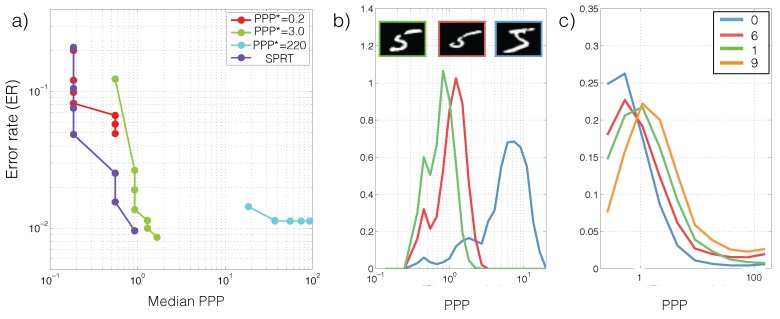
Low-light classification performance. (**a**) Error rate *vs*. PPP tradeoff for the SPRT algorithm (Equation (3)). $PPP^{*}=x$ denotes a “specialist”: A model trained using images only at light level *x* and tested on other light levels by input normalization (see Section 2.2.2). (**b**) SPRT decision time is stochastic even for the same underlying image. The PPP distribution is plotted separately for multiple images of 5. (**c**) SPRT decision time distribution is category-dependent. Some categories, e.g., “0”, are easier (faster decision) than others, say “9”.

Essentially, SPRT keeps accumulating photons by increasing exposure time until there is predominant evidence in favor of a particular category. Due to the stochasticity of the photon arrival events and the variability in an object’s appearance, the algorithm observes a different stream of photon counts each time. As a result, the exposure time *T*, and equivalently, the required PPP, are also different each time (see Figure 3).

The accuracy of the algorithm is controlled by the threshold *θ*. When a decision is made, the declared class $c^{*}$ satisfies that $\log\frac{P(Y=c^{*}|X_{1:t})}{P(Y\neq c^{*}|X_{1:t})}>\theta$, which means that class $c^{*}$ has at least posterior probability $Sigm\left(\theta \right)\overset{▵}{\;=}\frac{1}{1+e^{-}\theta }$ according to the generative model, and the error rate of SPRT is at most 1-Sigm(θ). For instance, if the maximum tolerable error rate is 10%, *θ* should be set so that 1-Sigm(θ)=0.1, or θ≈2.2, while an error rate of 1% would drive *θ* to 4.6. Since higher thresholds lead to longer exposure times, the threshold serves as a knob to trade off speed *versus* accuracy, and should be set appropriately based on *γ* (Equation (2)).

The assumption that the conditional distribution $P(Y=c|X_{1:t})$ is known is rather restrictive. Fortunately, the conditional distribution may be directly learned from data. In particular we train a recurrent neural network [13] $f_{c}\left(X_{1:t}\right)\approx P\left(Y=c|X_{1:t}\right)$ to approximate the conditional distribution. This network has a compact representation, and takes advantage of the sparseness of the photon-counts for efficient evaluation. Details of the network may be found in Section 3.2 and [9].

#### 2.2.2. Experiments

We evaluate the low-light classification performance of the SPRT on the MNIST dataset [11], a standard handwritten digits dataset with 10 categories. The images are 28×28 in resolution and in black and white. We simulate the outputs from a photon-counting sensor according to the full noise model (Section 3.1). The images are stationary within the imaging duration. We do not assume a given conditional distribution $P(Y|X_{1:t})$ but train a recurrent network approximation $f(X_{1:t})$ from data (Section 3.2).

**Figure 4:**
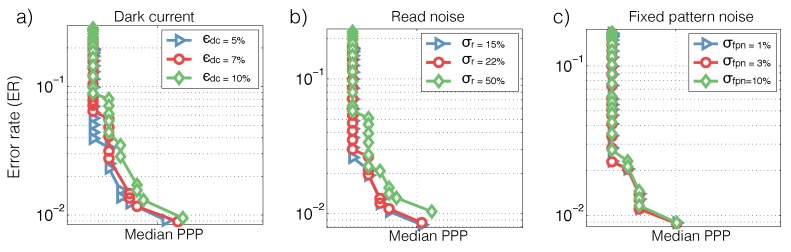
Sensitivity to noise in image classification. Error rate *vs*. PPP tradeoff with different levels of (**a**) dark current $\epsilon_{dc}$, (**b**) read noise and (**c**) fixed pattern noise (see Section 3.1). The default setting uses 3% dark current, 0% read noise and 0% fixed pattern noise.

Recall that classification correctness in each trial and the required exposure time (or PPP) are random variables. We therefore characterize SPRT performance based on the tradeoff between error rates (ER, $E[\hat{Y}\neq Y]$) and the median PPP in Figure 3a. The tradeoff is generated by sweeping the thresholds θ∈[-2.2,9.2]. For comparison we tested the performance of models that were trained to classify images from a single PPP. We call these models “specialists” for the corresponding PPP. The specialists are extended to classify images at different light levels by scaling the image to the specialized PPP. To get a sense of the intraclass and interclass PPP variability, we also visualize the PPP histograms for multiple runs of different images in the same class (Figure 3b), and the overall PPP histograms for a few classes (Figure 3c). Lastly, we analyze how SPRT’s performance is sensitive to sensor noises in Figure 4. Details of the analysis procedure are found in Section 3.1.

### 2.3. Search

Search is a generalization of classification into multiple locations. The task is to identify whether a target object (e.g., keys, a pedestrian, a cell of a particular type) is present in a scene cluttered with distractors (e.g., a messy desk, a busy street at night or a cell culture). Note that despite the multiple candidate positions for a target to appear, we consider search as a binary task, where the two hypotheses are denoted C=1 (target-present) and C=0 (target-absent). We assume for simplicity that at most one target may appear at a time (for multiple targets, see [14]).

The difficulty of search in low-light conditions may be attributed to the following factors. (1) There are multiple objects in the display, and each object is subject to photon count fluctuations. (2) Long range constraints, such as the prior knowledge that at most one target is present in the visual field, must be enforced. (3) Properties of the scene, such as the amount of clutter in the scene and the target and distractor appearance, may be uncertain. For example, we may know that there may be either three or twelve objects in the scene, and intuitively the search strategy for these two scenarios should be drastically different. Therefore, scene properties must be inferred for optimal performance.

We assume that a visual field consists of *L* non-overlapping locations, out of which *M* locations may contain an object. *M* represents the amount of clutter in the scene. The objects are simplified to be oriented bars and the only feature that separates a target from a distractor is the orientation. The orientation at location *l* is denoted $Y^{\left(l\right)}$. The target orientation and the distractor orientation are denoted $y_{T}$ and $y_{D}$, respectively. The scene properties are collected denoted $\phi =\{M,y_{T},y_{D}\}$. The scene properties may be unknown for many search tasks, thus *φ* is a vector of random variables. The variable of interest is C∈{0,1}: C=1 iff $\exists l\in \left\{1,\ldots ,L\right\},Y^{\left(l\right)}=y_{T}$, (*i.e.*, C=1 iff there exists a location that contains a target).

We also assume that a low-light classifier discussed in Section 2.2 has been developed for classifying bar stimulus: the classifier computes $f_{y}\left(X_{1:t}^{\left(l\right)}\right)\approx P\left(Y^{\left(l\right)}=y|X_{1:t}^{\left(l\right)}\right)$, the probability that the bar orientation at location *l* is *y* conditioned only on the local photon counts $X_{1:t}^{\left(l\right)}$.

#### 2.3.1. Search Algorithm

Similar to the low-light classification problem, an asymptotically optimal search algorithm is based on SPRT. The detailed algorithm is [12,14]:

(4) $$\begin{array}{cc} & \text{Choose two error thresholds}\;\;\;\theta_{0}<0,\theta_{1}>0\\ & \text{Compute}\;S\left(t\right)\overset{▵}{\;=}\log\frac{P(C=1|X_{1:t})}{P(C=0|X_{1:t})}\\ & \left\{\begin{array}{cc} \text{report}\;\;\;C=1 & \text{if}\;\;\;S\left(t\right)>\theta_{1}\\ \text{report}\;\;\;C=0 & \text{if}\;\;\;S\left(t\right)<\theta_{0}\\ \text{increase}\;\;\;t & \text{otherwise} \end{array}\right. \end{array}$$

where S(t) is the log likelihood ratio between the two competing hypotheses, target-present (C=1) and target-absent (C=0). This algorithm is a binary version of the classification algorithm in Equation (3). Similar to Equation (3), the two thresholds $\theta_{0}$ and $\theta_{1}$ controls the amount of false reject errors (*i.e.*, declare target-absent when target-present) and false accept errors (*i.e.*, declaring target-present when target-absent).

The key for SPRT is to compute S(t) from photon counts $X_{1:t}$. The inference procedure may be implemented by two circuits, one infers the scene properties *φ*, and the other computes S(t) (see [14]):

(5) $$\begin{array}{cc} S(t) & =\log\frac{1}{L}\sum_{l,\phi }R^{\left(l,\phi \right)}\left(X_{1:t}^{\left(l\right)}\right)P\left(\phi |X_{1:t}\right) \end{array}$$

where

(6) $$\begin{array}{cc} R^{\left(l,\phi \right)}\left(X_{1:t}^{\left(l\right)}\right) & \overset{▵}{\;=}\frac{\sum_{y}f_{y}\left(X_{1:t}^{\left(l\right)}\right)P\left(Y^{\left(l\right)}=y|\phi ,C^{\left(l\right)}=1\right)}{\sum_{y}f_{y}\left(X_{1:t}^{\left(l\right)}\right)P\left(Y^{\left(l\right)}=y|\phi ,C^{\left(l\right)}=0\right)} \end{array}$$

(7) $$\begin{array}{cc} P(\phi |X_{1:t}) & \propto P\left(\phi \right)\prod_{l}\sum_{y}f_{y}\left(X_{1:t}^{\left(l\right)}\right)\frac{P(Y^{\left(l\right)}=y|\phi ,C^{\left(l\right)}=0)}{P(Y^{\left(l\right)}=y)} \end{array}$$

Therefore, S(t) may be computed by composing the low-light classifiers $f_{y}\left(X_{1:t}^{\left(l\right)}\right)$ according to Equations (5)–(7). The probabilities used in Equations (6) and (7), such as $P(Y^{\left(l\right)}$ = y) and $P(Y^{\left(l\right)}$ = y|ϕ,C=2), may be estimated from past data.

#### 2.3.2. Experiments

**Figure 5:**
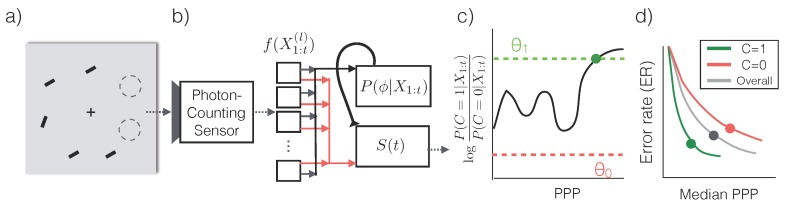
low-light Search. (**a**) Search stimuli with L=7 display locations and 5 objects (oriented bars). The dashed circles are not part of the display but used to indicate empty locations. (**b**) The search algorithm. Local classification results $f(X_{1:t}^{\left(l\right)})$ go through two circuits, one estimates the scene properties $P(\phi |X_{1:t})$ (Equation (7)) and sends feedback to the other circuit that computes the log likelihood ratio S(t) (Equation (5)). (**c**) SPRT compares S(t) against a pair of thresholds $\theta_{1}$ and $\theta_{0}$ to decide whether to declare target-presence or absence, or wait for more evidence (Equation (4)). (**d**) SPRT produces ER *vs*. PPP tradeoffs (sketch) for different conditions.

**Figure 6:**
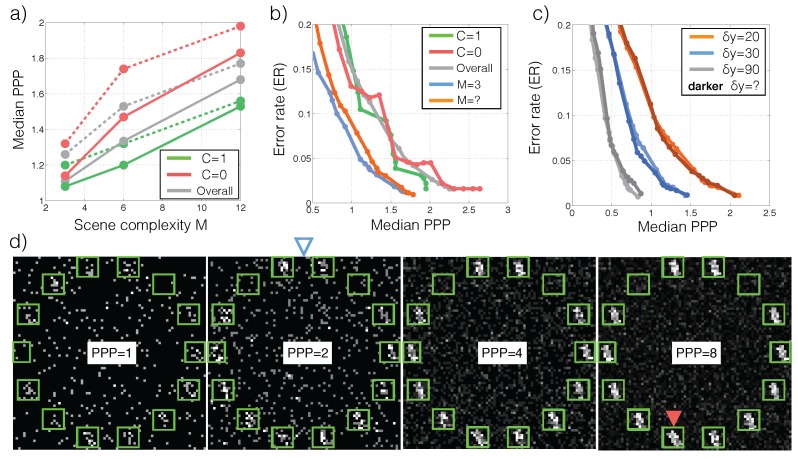
Characteristics of the search algorithm. (**a**) Median PPP required to achieve 5% error rate as a function of scene complexity *M*. Solid/dashed lines represent search problems where the complexity is known/unknown in advance. (**b**) ER *vs*. PPP tradeoff for various conditions. The first three legends corresond to target-present (C = 1), target-absent (C = 0) and their average for M=12. The last two corresponds to a simpler image containing three bars with the complexity known (M=3) and unknown (M=?) in advance. (**c**) ER *vs*. PPP tradeoff for different appearance differences $\delta y=|y_{T}-y_{D}|$ between the target and the distractor. Darker lines denote the cases where the difference is unknown before hand. (**d**) Examples of lowlight search stimuli as a function of PPP. L=14, M=12, $\delta y=20^{\circ }$, target-present (C = 1, location indicated by red arrow, same for every PPP). The photon-counting sensors receive inputs from within the green windows. With known complexity the algorithm achieves a 1% error rate with a median PPP of 2 (blue hollow arrow).

We choose a simple setup (Figure 5a) to illustrate how the performance of the search algorithm is affected by scene properties: the amount of clutter *M*, the target/distractor appearances $y_{T}$ and $y_{D}$, as well the degree of uncertainty associated with them. The setup contains L=14 locations, each occupying a 7×7 area from which the sensor collects photons. The area contains a 3×7-pixel bar with intensity 1 and background pixels with intensity 0. The max emission rate is $\lambda_{max}=3$ photons/s, and the dark current is 50% (causing the background to emit 1 photon/s). Examples of the lowlight search setup are shown in Figure 6d.

We conduct two experiments, one manipulates the scene complexity *M* and the other target/distractor appearances. In the first experiment *M* is either chosen uniformly from {3,6,12}, or fixed at one of the three values (Figure 6a,b): (1) Despite the high dark current noise, a decision may be made quickly with less than 2 photons per pixel. (2) The amount of light required to achieve a given classification error increases as *M*. (3) Not knowing the complexity further increases the required photon count. (4) Target-absent conditions requires more photons than target-present conditions. In the second experiment the target-distractor appearance difference $\delta y=|y_{T}-y_{D}|$ is either chosen uniformly from $\left\{20^{\circ },30^{\circ },90^{\circ }\right\}$ or fixed at one of the three. Figure 6c suggests that target dissimilarity heavily influences the ER-PPP tradeoff, while uncertainty in the target and distractor appearances does not.

### 2.4. Tracking

Finally, we demonstrate the potential of photon-counting sensors in tracking under low-light conditions. The goal of tracking is to recover time-varying attributes (*i.e.*, position, velocity, pose, *etc.*) of one or multiple moving objects. It is challenging because, unlike classification and search, objects in tracking applications are non-stationary by definition. In low-light environments, as the object transitions from one state to another, it leaves only a transient footprint, in the form of stochastically-sprinkled photons, which is typically insufficient to fully identify the state. Instead, a tracker must postulate the object’s dynamics and integrate evidence over time accordingly. The evidence in turn refines the estimates of the dynamics. Due to the self-reinforcing nature of this procedure, the tracker must perform optimal inference to ensure convergence to the true dynamics.

Another challenge that sets low-light tracking apart from regular tracking problems is that the observation likelihood model is not only non-Gaussian, but also often unavailable, as it is commonly the case for realistic images. This renders most Kalman filter algorithms [15] ineffective.

#### 2.4.1. Tracking Algorithm

The tracking algorithm we have designed is a hybrid between the Extended Kalman Filter [15] and the Auxiliary Particle Filter [16]. Let $Z_{t}$ denote the state of the object, *F* the forward dynamics that govern the state transition: $Z_{t+1}=F\left(Z_{t}\right)$, which are known and differentiable, and $P_{t}\left(Z\right)$ the posterior distribution over the states at time *t*: $P_{t}\left(Z\right)\overset{▵}{\;=}P\left(Z_{t}=Z|X_{1:t}\right)$.

We make two assumptions: (1) $P_{t}$ may be approximated by a multivariate Gaussian distribution; (2) A low-light regressor $f\left(Z|X_{t}\right)\approx P\left(Z_{t}=Z|X_{t}\right)$ is available to compute a likelihood score of $Z_{t}$ given only the snapshot $X_{t}$ at time *t*. $f(Z|X_{t})$ does not have to be normalized. We justify assumption (1) and describe algorithms for realizing assumption (2) in Section 3.3. As we will see in Equation (17), the Poisson noise model (Equation (1)) ensures that $f(Z|X_{t})$ exists and takes a simple form.

**Figure 7:**
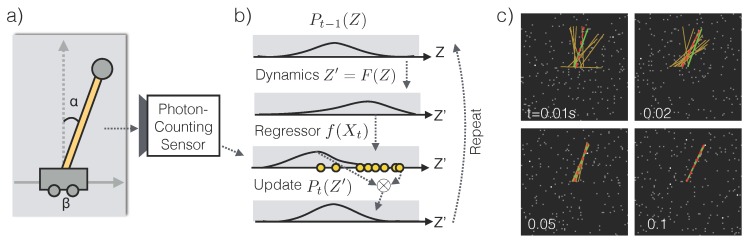
Low-light tracking. (**a**) An illustration of an inverted pendulum with attributes of interest *α* (pendulum angle) and *β* (cart location). The pole (yellow) is bright and everything else is dark. (**b**) Tracking algorithm (Equation (8)) iteratively updates the posterior $P_{t}\left(Z\right)$ using new evidence $X_{t}$ and a low-light regressor $f(X_{t})$. (**c**) Snapshots of a sample run at exposure times t=0.01,0.02,0.05 and 0.1 s. The brightest pixels emit photons at $\lambda_{max}=10$ photons/s and the dark current $\epsilon_{dc}=50\%$. The true position of the pendulum pole is shown in green, its estimate in red dashed, and samples from the tracker’s posterior in yellow.

Given a prior probability distribution $P_{0}$, our goal is to compute the posterior distribution $P_{t}$ for all *t*. The tracking algorithm starts with t=0 and repeat the following procedure (Figure 7b).

(8) $$\begin{array}{cc} & 1.\;\text{Compute the predictive distribution}\;P\left(Z_{t+1}|Z_{t}\right)\;\text{from}\;P_{t}\\ & 2.\;\text{Draw}\;K\;\text{samples}\;Z_{s}^{\prime }\;\text{from}\;P\left(Z_{t+1}|Z_{t}\right)\\ & 3.\;\text{Observe}\;X_{t+1}\;\text{and compute}\;W_{s}=f\left(Z_{s}^{\prime }|X_{t+1}\right)\\ & 4.\;\text{Approximate}\;P_{t+1}\left(Z^{\prime }\right)\;\text{as using samples}\;Z_{s}^{\prime }\;\text{weighted by}\;W_{s}\\ & 5.\;\text{Increase}\;t=t+1 \end{array}$$

Under the Gaussian assumption for $P_{t}$, both steps 1 and 4 may be computed in close-form (Section 3.3). This is in sharp contrast to regular particle filters, which do not assume any parametric form for $P_{t}$ and accomplish steps 1 and 4 using samples. Empirically we found that the Gaussian assumption is reasonable and often leads to efficient solutions with less variability.

#### 2.4.2. Experiments

We choose the 1D inverted pendulum problem (Figure 7a) that is standard in control theory. A pendulum is mounted via a a massless pole on a cart. The cart can move horizontally in 1D on a frictionless floor. The pendulum can rotate full circle on a fixed 2D plane perpendicular to the floor. The pendulum is released at time t=0 at an unknown angle $\alpha_{0}\in \left[0,360^{\circ }\right)$ from the vertical line, while the cart is at an unknown horizontal offset $\beta_{0}\in R$. The task is to identify how the angle $\alpha_{t}$ and the offset $\beta_{t}$ change through time from the stream of photon counts $X_{1:t}$. The state of the pendulum system is $Z_{t}=\left\{\alpha_{t},{\dot{\alpha }}_{t},\beta_{t},\dot{\beta_{t}}\right\}$. The system’s forward dynamics is well-known [17].

In our simulations, only the pole of the pendulum is white and everything else is dark. The highest photon emission rate of the scene is $\lambda_{max}$ and the dark current rate is $\epsilon_{dc}$. We systematically vary $\lambda_{max}$ and $\epsilon_{dc}$ and observe the amount of estimation error in the angle $\alpha_{t}$ and cart position $\beta_{t}$. See Section 3.4 for the simulation procedure.

**Figure 8:**
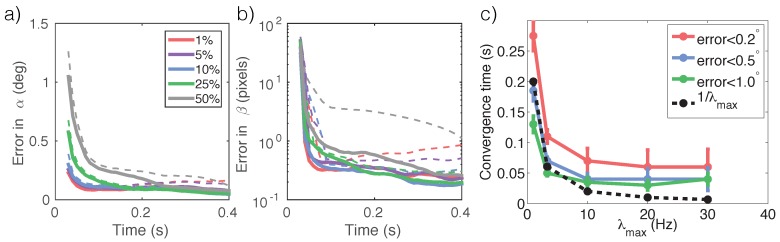
Tracking performance. The average estimation error in (**a**) the pendulum angle $\alpha_{t}$ and (**b**) in cart position $\beta_{t}$ over time as a function of the amount of dark current $\epsilon_{dc}$ (color-coded). The photon emission rate is set at $\lambda_{max}=10$ Hz. Dashed lines shows 1std above the mean. (**c**) The tracker’s convergence times for its angle estimates to be within $0.2^{\circ },0.5^{\circ }$ and $1^{\circ }$, respectively, of the truth as a function of illuminance. $\epsilon_{dc}$ is set at 10%. As a reference “$1/\lambda_{max}$” is inversely proportional to the illuminance and scaled to have roughly the same starting position as the “error $<0.5^{\circ }$” curve.

We see that (1) estimation errors decrease over time (Figure 8a,b), (2) smaller $\epsilon_{dc}$ leads to faster reduction in estimation error on average (Figure 8a,b), and (3) the tracker’s *convergence time*, *i.e.*, the time it takes to achieve a certain level of estimation accuracy, decreases with illuminance (Figure 8c). The time required to satisfy high accuracy requirements (e.g., <1${}^{\circ }$ for *α* estimation) does NOT follow a simple inverse proportional relationship with illuminance. Instead, the convergence time plateaus, potentially due to the noise in the sampling procedure (Equation (8), step 2).

## 3. Materials and Methods

### 3.1. Imaging Model Including Noise Sources

Within an interval δt, the sensor readout *x* is corrupted by a series of noise sources.

1. The amount of photons *N* incident on the pixel is subject to Poisson noise (shot noise). The noise level is determined by the true intensity *I* and the dark current $\epsilon_{dc}∼N\left(0,\sigma_{\epsilon }\right)$.
2. The photon counts are corrupted by an additive Gaussian read noise $\epsilon_{r}∼N\left(0,\sigma_{r}\right)$ and a multiplicative fixed pattern noise $\epsilon_{fpn}∼N\left(0,\sigma_{fpn}\right)$.

(9) $$\begin{array}{r} N∼Poisson(\cdot |I+\epsilon_{dc}) \end{array}$$

(10) $$\begin{array}{r} x=max(0,\left(N+\epsilon_{r}\right)\left(1+\epsilon_{fpn}\right)) \end{array}$$

Sensors designed for low light applications (e.g., [5]) have promised low read noise and low fixed pattern noise. Therefore, we focus on modeling the shot noise and dark current, and assume that algorithms have access to *N* when the algorithms are trained. We then test the algorithm using realistic values of read noise and fixed pattern noise to study robustness against noise (see Figure 4).

### 3.2. Low-Light Classifier

A low-light classifier $f(X_{1:t})$ that approximates the conditional distribution $P(Y|X_{1:t})$ for *Y* = {1,2,…,C} is developed in [9]. The classifier is a recurrent neural network consisting of multiple layers $h^{\left(1\right)}\left(t\right),\ldots ,h^{\left(L\right)}\left(t\right)$ where the activation at layer *l* is:

(11) $$\begin{array}{cc} h_{j}^{\left(l\right)}\left(t\right) & =\sum_{\tau =1}^{t}W_{j}^{\left(l\right)}X_{\tau }+b_{i}t \end{array}$$

(12) $$\begin{array}{cc} & =h_{j}^{\left(l\right)}\left(t-1\right)+W_{j}^{\left(l\right)}X_{t}+b_{i} \end{array}$$

where $W_{j}^{\left(l\right)}\in R^{d}$ and $b_{j}^{\left(l\right)}\in R$ are the weights and the biases of the *j*-th unit. Equation (12) suggests that $h_{j}^{\left(l\right)}\left(t\right)$ may be computed incrementally from its old value $h_{j}^{\left(l\right)}\left(t-1\right)$.

The hidden units at layer *l* are organized into non-overlapping groups and pooled. A pooling unit $h_{k}^{\left(l\right)}$ oversees the hidden units at block $G_{k}$, and its activation is computed by:

(13) $$\begin{array}{c} m_{k}^{\left(l\right)}\left(t\right)=\max(0,\max_{j\in G_{k}}\left(h_{j}^{\left(l\right)}\left(t\right)\right) \end{array}$$

Let $j^{*}\left(t\right)=\arg\max_{j\in G_{k}}\left(h_{j}^{\left(l\right)}\left(t\right)\right)$ denote the index of the max unit at time t-1 ($j^{*}\left(t\right)=0$ denote the event that the max value is 0). If within time interval δt only a small set $G_{k}^{\prime }\subseteq G_{k}$ of hidden units within group $G_{k}$ are updated, $m_{k}^{\left(l\right)}\left(t\right)$ may also be computed incrementally:

(14) $$\begin{array}{c} m_{k}^{\left(l\right)}\left(t\right)=\max_{j\in G_{k}^{\prime }\cup j^{*}\left(t-1\right)}\left(h_{j}^{\left(l\right)}\left(t\right)\right) \end{array}$$

Both Equations (12) and (14) are critical for an efficient implementation of the classifier. For example, if only a tenth of the units are updated in each layer, the computation time for $f(X_{1:t})$ may be reduced by a factor of 10.

Finally, the output of the classifier is given by:

(15) $$\begin{array}{cc} f_{c}\left(X_{1:t}\right) & =\frac{\exp(m_{c}^{\left(L\right)}\left(t\right))}{\sum_{c^{\prime }}\exp\left(m_{c^{\prime }}^{\left(L\right)}\left(t\right)\right)},\;\;\;c\in \left\{1,2,\ldots ,C\right\} \end{array}$$

Since $f(X_{1:t})$ approximates the conditional likelihood $P(Y=c|X_{1:t})$, the parameters ${\left\{W_{j}^{\left(l\right)},b_{j}^{\left(l\right)}\right\}}_{j,l}$ of f(·) may be learned by maximum likelihood from a dataset of photon counts. However, it is expensive to keep track of a high number of photon count streams. Fortunately, Equation (11) also suggests that the network’s prediction at time *t* only depends on the cumulative photon counts $S_{t}\overset{▵}{\;=}\sum_{\tau =1}^{t}X_{\tau }$. Therefore, one only needs a dataset of $\left\{S_{t},t,Y\right\}$ tuples to perform maximum likelihood learning.

In detail, we simulated a lowlight MNIST dataset ${\left\{S^{\left(i\right)},PPP^{\left(i\right)},Y^{\left(i\right)}\right\}}_{i}$ where the PPPs are sampled uniformly from {0.22,2.2,22,220}. Note that we are using PPP instead of the exposure time *t* for reasons discussed in Section 2.1. At PPP=220 each image pixel contains around 5 bits of information (log signal-to-noise-ratio ≈5). Our implementation uses the MatConvNet package [18] and its default hyper-parameters for training. We train a model with the same connectivity as the LeNet [11] denoted: 784-20-50-500-10. The model contains 784 input units, followed by two convolutional hidden layers with 20 and 50 filters, respectively, of size 5×5. Inputs to a convolutional layer is convolved with the filters, and then pooled over 2×2 non-overlapping windows. After the convolutional layers are a fully connected hidden layer with 500 units and a fully connected softmax layer with 10 output categories. We minimize the negative log likelihood with a L2 weight decay:

(16) $$\begin{array}{c} -\sum_{i}\log f_{Y^{\left(i\right)}}\left(S^{\left(i\right)}\right)+\eta \sum_{l}\left|\right|W^{\left(l\right)}{\left|\right|}_{2}^{2} \end{array}$$

where η=0.0005 is the strength of the weight decay. We use stochastic gradient descent with mini-batches of 100 and train for 60 epochs with learning rate =0.001 and momentum =0.9.

### 3.3. Tracking Algorithm

#### 3.3.1. Low-Light Regressor

The photon count $X_{t}$ at time *t* depends only on the angle $\alpha_{t}$ and cart position $\beta_{t}$ and not their time derivatives, so our low-light regressor can only predict $\alpha_{t}$ and $\beta_{t}$: $f\left(Z_{t}|X_{t}\right)\;=\;f\left(\alpha_{t},\beta_{t}|X_{t}\right)$. Since we simulate the scene using a generative model, we can compute the exact form of the low-light regressor. Let $P(I_{t}|\alpha_{t},\beta_{t})$ be the generative model where $I_{t}$ is the intensity value of the image, the photon emission rate $\lambda_{i}$$\left(\alpha_{t},\beta_{t}\right)$ for every pixel may be computed using Equation (1). As a result the log likelihood of observing $X_{t}$ is:

(17) $$\begin{array}{cc} \log P(X_{t}|\alpha_{t},\beta_{t}) & =\log\prod_{i}Poisson\left(X_{ti};\lambda_{i}\left(\alpha_{t},\beta_{t}\right)\right)=Const.+X_{t}^{T}\log\left(\lambda \left(\alpha_{t},\beta_{t}\right)\right)-1^{T}\lambda \left(\alpha_{t},\beta_{t}\right) \end{array}$$

Note that this likelihood model is linear in the intensity image *λ* given the parameters $\alpha_{t}$ and $\beta_{t}$, and *not* linear in terms of the parameters themselves. In addition, the likelihood is Poisson, not Gaussian. Hence Kalman filters are not applicable here.

Using Bayes rule we have:

(18) $$\begin{array}{c} f\left(Z_{t}|X_{t}\right)=P\left(\alpha_{t},\beta_{t}|X_{t}\right)\propto P\left(X_{t}|\alpha_{t},\beta_{t}\right)P\left(\alpha_{t},\beta_{t}\right) \end{array}$$

When a generative model is not available, the regressor may be trained discriminatively on a dataset using maximum likelihood, similar to Section 3.2.

#### 3.3.2. Approximating the Predictive Distribution (Step 1 of Equation (8))

Let $P_{t}\approx N\left(\mu_{t},\Sigma_{t}\right)$ and *F* be the dynamics of the inverted pendulum. The predictive distribution may be approximated as a gaussian by linearizing *F*:

(19) $$\begin{array}{c} Z_{t+1}|Z_{t}\approx N\left(F\left(\mu_{t}\right),\left(▿F{|}_{\mu_{t}}\right)\Sigma {\left(▿F{|}_{\mu_{t}}\right)}^{T}\right) \end{array}$$

where ${▿F|}_{\mu_{t}}$ is the Jacobian of the dynamics *F* evaluated at the prior mean $\mu_{t}$.

#### 3.3.3. Approximating the Posterior Distribution (Step 4 of Equation (8))

Given weighted samples ${\left\{Z_{s}^{\prime }\right\}}_{s=1}^{K}$ and normalized weights ${\left\{W_{s}\right\}}_{s=1}^{K}$ (*i.e.*, $\sum_{s}W_{s}=1$), the posterior $P_{t+1}$ may be approximated by a Gaussian with: $P_{t+1}\approx N\left(\mu_{t+1},\Sigma_{t+1}\right)$, where

(20) $$\begin{array}{ll} \mu_{t+1} & =\sum_{s}W_{s}Z_{s}^{\prime } \end{array}$$

(21) $$\begin{array}{ll} \Sigma_{t+1} & =\frac{\sum_{s}W_{s}\left(Z_{s}^{\prime }-\mu_{t+1}\right){\left(Z_{s}^{\prime }-\mu_{t+1}\right)}^{T}}{1-\sum_{s}W_{s}^{2}} \end{array}$$

### 3.4. Tracking Experiment

The pole of the pendulum has an intensity of 1 while everything else (background, cart, *etc*) has intensity 0. The pole is 3×30 pixels and the entire scene is 80×80 pixels. The pendulum is half the mass of the cart. The maximum photon emission rate is $\lambda_{max}$, and dark current is $\epsilon_{dc}$. The pendulum is released at the origin ($\beta_{0}=0$) with $\alpha_{0}$ randomly chosen from $\left\{10^{\circ },20^{\circ },30^{\circ },40^{\circ }\right\}$. Each initial state is simulated 25 times and then aggregated, yielding a total of 100 trials for each condition (*i.e.*, for each pair of $\lambda_{max}$ and $\epsilon_{dc}$). The distribution on $\alpha_{0}$ and $\beta_{0}$ used for simulation is not available to the tracking algorithm, which instead assumes uniform distributions for both.

#### 3.4.1. Internal Noise Due to Sampling

For Figure 7a,b we used K=1000 samples from the predictive distribution (Equation (8), step 2). Repeating the experiment using K=300 samples shows the same trend with larger error bars. One counterintuitive finding is that as the exposure time increases, the estimation error first decreases and then diverges (both higher mean and deviation are visible, Figure 7a,b). The degree of divergence is aggravated by increasing signal-to-noise ratio (or reducing dark current $\epsilon_{dc}$). This may be explained by the internal noise in the tracking algorithm. The algorithm relies on samples from the predictive distribution $P(Z_{t+1}|Z_{t})$ (Equation (8), step 1) to coincide with states that have a high observation likelihood $f(Z|X_{t})$ (Equation (8), step 3). This coincidence is less likely to happen when the observation likelihood becomes precise, or as the posterior becomes sharply-peaked. Similarly, for Figure 7c we used K=1000, and repeated the experiment with K=300. They obtain exactly the same trends except that smaller *K* corresponds to a higher plateau for the convergence time (Figure 7c), indicating that sampling noise may be limiting the speed for accurate tracking.

## 4. Discussion and Conclusions

The advent of photon-counting sensors motivates us to reconsider the prevalent paradigm in computer vision: Rather than first capturing an image and then analyzing it, we should design algorithms that incrementally compute information from the stream of photons that hits the sensor, without any attempt to reconstruct the image. This style of thinking is particularly attractive in low light conditions, where the exposure time required for capturing a high-quality image is prohibitively lengthy.

Photon-counting sensors deliver small increments of the image at short delays and high frequencies. We show that this incremental input could in principle be applied to solve a variety of vision problems with a short exposure time. Algorithms that are inspired by the asymptotically optimal SPRT appear particularly well suited for minimizing photon counts while satisfying a desired accuracy bound.

Our first finding is that useful information may be computed in a short amount of time, well ahead of the integration time that is required for forming (or reconstructing) a high quality image. In Figure 1b,c, we see that a low-light classification algorithm can achieve 1% classification error of handwritten digits before one photon per pixel has been collected. The low-light classifier may be viewed as reconstructing the features (instead of the image), and carrying the uncertainty of the features all the way to classification. This uncertainty is essential in a sequential decision making setting to determine when to stop collecting more photons. In comparison, conventional approaches simply reconstruct the image, and pass it to a classifier trained on high-quality images. The conventional approach suffers from two issues. (1) Since the conventional approach discards the uncertainty information, it is not clear how to determine the required exposure time; and (2) statistics of the reconstruction may be different from that of high-quality images, hence the classifier’s performance may not be guaranteed.

Second, algorithms for classification and search from streams of photons are photon-efficient: they stop as soon as a confident decision is made. This efficiency is critical for domains such as astrophysics where each photon is precious [19], and cell imaging applications where the dies that are employed to visualize cell structures are phototoxic [20,21]. As an example of the photon efficiency, Figure 3a shows that at PPP =1 the low-light classifier based on SPRT can already achieve a better performance than a classifier using PPP =220. Additionally, contrary to the conventional paradigm that obtains images with a fixed duration, low-light classifiers and search algorithms uses different exposure times depending on the specific photon arrival sequence (Figure 3b) and on the overall classification difficulty of the example (Figure 3b,c).

Third, algorithms become faster when more light is present. For classification and search where the input image is stationary, time is synonymous with the amount of photons. Higher illuminance therefore translates to faster decisions. This simple relationship is useful in that a low-light system trained for classification or search at one illuminance level may be easily applied at another illuminance level. The transition only requires knowing the illuminance level of the new scene, which may be estimated either via an explicit illuminance sensor or from the total photon count across the image [22]. In addition, the ER *vs*. median PPP tradeoff (Figure 3a and Figure 6a–c) is an illuminance-independent characteristic of the algorithm and the task.

Last, the relationship between illuminance and speed is not always simple in tracking. The dynamics governing the object movement/state transition has its own time scale. A regressor $f(X_{t})$ thus has only a finite duration for integrating information before the object moves too far. A tracker relies on accurate prediction of the regressor to postulate the object’s next position. An inaccurate prediction due to short exposure time may cause tracking failure. In addition, internal noise in the tracking algorithm (Section 3.4) may cause the speed to plateau after a certain illuminance level. As a result, the relationship between illuminance and convergence time is not a simple inversely proportional relationship, as shown in Figure 8c.

Although we have provided proof-of-concept illustrations of low-light vision applications with photon-counting sensors, many challenges still remain. (1) We are not aware of any hardware specialized at processing streams of photon counts at high speeds. Nonetheless, current Field-programmable Gate Array (FPGA) implementations have achieved over 2000 Hz throughput for classifying images of a similar resolution as those in Section 2.2 [23]. In addition, the low-light classifiers implemented as a recurrent neural network (Section 2.2) can be updated incrementally, *i.e.*, $f(X_{1:t})$ can be computed from the internal states of $f(X_{1:t-1})$ and $X_{t}$ with sparse updates. The sparseness may be key to expedite computation. (2) We do not yet have datasets collected directly from photon-counting sensors to verify the robustness of the proposed methodology, as many such sensors are still in the making [5,6,7]. (3) Our noise model (Section 3.1) may be a crude approximation to handle moving objects. For example, we do not model motion induced blur or input disturbances due to camera self-motion. Nonetheless, motion induced blur may not be an issue if the sensor is collecting a single photon at a time, such as in low light and/or high-frequency imaging scenarios. In these scenarios even though the amount of photons is so low that full image reconstruction is difficult, our algorithm can still make correct and full use of the where and when of photon arrivals. This is precisely the advantage of image-free vision.

In conclusion, we propose to integrate computer vision with photon-counting sensors to address the challenges facing low-light vision applications. We should no longer wait for a high-quality image to be formed before executing the algorithm. Novel algorithms and hardware solutions should be developed to operate on streams of photon-counts. These solutions should also sidestep image reconstruction and focus directly on the task at hand.

## Abbreviations

The following abbreviations are used in this manuscript:

CPU: Central Processing Unit
ER: Error rates
FPGA: Field-programmable Gate Arrays
MNIST: The Mixed National Institute of Standards and Technology dataset
PPP: The number of photons per pixel in a low-light image, averaged across all locations and the imaging duration
SPRT: Sequential probability ratio test

## References

[1] Hall E., Brenner D. (2014). Cancer risks from diagnostic radiology. Cancer.

[2] Stephens D.J., Allan V.J. (2003). Light microscopy techniques for live cell imaging. Science.

[3] Brida G., Genovese M., Berchera I.R. (2010). Experimental realization of sub-shot-noise quantum imaging. Nat. Photonics.

[4] Zappa F., Tisa S., Tosi A., Cova S. (2007). Principles and features of single-photon avalanche diode arrays. Sens. Actuators A Phys..

[5] Fossum E. The quanta image sensor (QIS): Concepts and challenges. Proceedings of the Computational Optical Sensing and Imaging 2011.

[6] Fossum E.R. Multi-Bit Quanta Image Sensors. Proceedings of the International Image Sensor Workshop.

[7] Sbaiz L., Yang F., Charbon E., Süsstrunk S., Vetterli M. The gigavision camera. Proceedings of the IEEE International Conference on Acoustics, Speech and Signal Processing.

[8] Morris P.A., Aspden R.S., Bell J.E., Boyd R.W., Padgett M.J. (2015). Imaging with a small number of photons. Nat. Commun..

[9] Chen B., Perona P. Scotopic Visual Recognition. Proceedings of the IEEE International Conference on Computer Vision Workshops.

[10] Abu-Naser A., Galatsanos N.P., Wernick M.N. (2006). Methods to detect objects in photon-limited images. JOSA A.

[11] LeCun Y., Bottou L., Bengio Y., Haffner P. (1998). Gradient-based learning applied to document recognition. IEEE Proc..

[12] Wald A. (1945). Sequential tests of statistical hypotheses. Ann. Math. Stat..

[13] Elman J.L. (1991). Distributed representations, simple recurrent networks, and grammatical structure. Mach. Learn..

[14] Chen B., Perona P. (2015). Speed versus accuracy in visual search: Optimal performance and neural architecture. J. Vis..

[15] Kalman R.E. (1960). A new approach to linear filtering and prediction problems. J. Fluids Eng..

[16] Pitt M.K., Shephard N. (1999). Filtering via simulation: Auxiliary particle filters. J. Amer. Stat. Assoc..

[17] Liberzon D. (2012). Switching in Systems and Control.

[18] Vedaldi A., Lenc K. MatConvNet—Convolutional Neural Networks for MATLAB. http://arxiv.org/abs/1412.4564.

[19] Martin D.C., Chang D., Matuszewski M., Morrissey P., Rahman S., Moore A., Steidel C.C. (2014). Intergalactic medium emission observations with the Cosmic Web Imager. I. The circum-QSO medium of QSO 1549+19, and evidence for a filamentary gas inflow. Astrophys. J..

[20] Hoebe R., Van Oven C., Gadella T.W., Dhonukshe P., Van Noorden C., Manders E. (2007). Controlled light-exposure microscopy reduces photobleaching and phototoxicity in fluorescence live-cell imaging. Nat. Biotechnol..

[21] Ji N., Magee J.C., Betzig E. (2008). High-speed, low-photodamage nonlinear imaging using passive pulse splitters. Nat. Methods.

[22] Cheng D., Price B., Cohen S., Brown M.S. Effective Learning-Based Illuminant Estimation Using Simple Features. Proceedings of the IEEE Conference on Computer Vision and Pattern Recognition.

[23] Ovtcharov K., Ruwase O., Kim J.Y., Fowers J., Strauss K., Chung E.S. Accelerating Deep Convolutional Neural Networks Using Specialized Hardware, Microsoft Research Whitepaper. http://research.microsoft.com/apps/pubs/?id=240715.

